# Dietary High Dose of Iron Aggravates the Intestinal Injury but Promotes Intestinal Regeneration by Regulating Intestinal Stem Cells Activity in Adult Mice With Dextran Sodium Sulfate-Induced Colitis

**DOI:** 10.3389/fvets.2022.870303

**Published:** 2022-06-15

**Authors:** Yitong Zhang, Lanmei Yin, Xianglin Zeng, Jun Li, Yuebang Yin, Qiye Wang, Jianzhong Li, Huansheng Yang

**Affiliations:** ^1^Hunan International Joint Laboratory of Animal Intestinal Ecology and Health, Laboratory of Animal Nutrition and Human Health, College of Life Sciences, Hunan Normal University, Changsha, China; ^2^Hunan Provincial Key Laboratory of Animal Nutritional Physiology and Metabolic Process, Scientific Observing and Experimental Station of Animal Nutrition and Feed Science in South-Central, Ministry of Agriculture, Hunan Provincial Engineering Research Center for Healthy Livestock and Poultry Production, Key Laboratory of Agro-ecological Processes in Subtropical Region, Institute of Subtropical Agriculture, Chinese Academy of Sciences, Changsha, China; ^3^National Center of Technology Innovation for Synthetic Biology, Tianjin Institute of Industrial Biotechnology, Chinese Academy of Sciences, Beijing, China; ^4^State Key Laboratory of Food Safety Technology for Meat Products, Yinxiang Group, Fujian Aonong BiologicaI Science and Technology Group Co., Ltd., Key Laboratory of Swine Nutrition and Feed Science of Fujian Province, Aonong Group, Zhangzhou, China

**Keywords:** high iron, dextran sodium sulfate-induced colitis, intestinal injury, intestinal repair, adult mice

## Abstract

The effects of excessive dietary iron intake on the body have been an important topic. The purpose of this study was to investigate the effects of high-dose iron on intestinal damage and regeneration in dextran sodium sulfate (DSS)-induced colitis model mice. A total of 72 8-week-old adult C57BL/6 mice were randomly divided into two dietary treatment groups: the basal diet supplemented with 45 (control) and 450 mg/kg iron (high-iron) from ferrous sulfate. The mice were fed different diets for 2 weeks, and then 2.5% DSS was orally administered to all mice for 7 days. Samples of different tissues were collected on days 0, 3, and 7 post administration (DPA). High-iron treatment significantly decreased the relative weight of the large intestine at 7 DPA but not at 0 DPA or 3 DPA. High dietary iron increased the jejunal villus width at 0 DPA, decreased the villus width and the crypt depth of the jejunum at 3 DPA, and decreased the number of colonic crypts at 7 DPA. Meanwhile, high dietary iron decreased the number of goblet cells in the jejunal villi and the Paneth cells in the jejunal crypts at 0 DPA, increased the number of goblet cells per crypt of the colon at 3 DPA, and the number of Paneth cells in the jejunal crypts, the goblet cells in the colon, the Ki67-positive proliferating cells in the colon, and the Sex-determining region Y-box transcription factor 9^+^ (SOX9) cells in the jejunum crypts and colon at 7 DPA. The organoid formation rate was increased by high-iron treatments at 3 DPA and 7 DPA. High dietary iron treatment decreased the mRNA level of jejunal jagged canonical Notch ligand 2 (*Jag-2*) at 0 DPA and bone morphogenetic protein 4 (*Bmp4*) and neural precursor cell-expressed developmentally downregulated 8 (*Nedd8*) in the jejunum and colon at 7 DPA, whereas it increased the mRNA expression of the serum/glucocorticoid-regulated kinase 1 (*Sgk1*) in the colon at 3 DPA. The results suggested that a high dose of iron aggravated intestinal injury but promoted intestinal repair by regulating intestinal epithelial cell renewal and intestinal stem cell activity in adult mice with colitis.

## Introduction

Inflammatory bowel disease (IBD) mainly includes Crohn's disease and ulcerative colitis (1). Iron deficiency is a common complication in patients with IBD, and patients are encouraged to eat a high-iron diet (2, 3). Some studies have shown that the chemical properties of iron put pressure on the intestines during inflammation (4). High-iron diets cause oxidative stress (5), inhibiting the growth, differentiation, and proliferation of most cells (6, 7). However, few studies have examined the effect of a high-iron diet on intestinal repair after injury in adult mice with colitis.

Animal models of colitis are induced using dextran sodium sulfate (DSS) (8). Symptoms include colon edema (9), rectal bleeding, diarrhea, and weight loss (10). In addition, the DSS-induced rat model of ulcerative colitis is accompanied by impaired jejunal barrier function (11). The regeneration phase of the injured intestine usually lasts 4 days after injury, and the intestinal tract returns to the normal stage 7 days after injury (12).

The integrity and regenerative potential of intestinal epithelial cells play key roles in fighting inflammation (13). A distinct feature of the intestinal morphology is the compartmentation of the epithelium into prominent villi composed of differentiated cells and the invagination of crypts containing stem and progenitor cells at their base. In mammals, the intestinal epithelium is the most active self-regenerative tissue (14) and is constantly renewed by intestinal stem cells (ISCs) located in the crypt bottoms (15). The continuous renewal and repair of the intestinal mucosal epithelium after injury depend on ISCs. ISCs are capable of differentiating into progenitor cells, and these newly formed cells proliferate and differentiate along the crypt-villus axis of the small intestine and colon (16), differentiating into one of the four main cell types (intestinal epithelial cells, goblet cells, Paneth cells, and intestinal endocrine cells). The villus height, crypt depth, and Ki67 expression have been used to determine cell proliferation (17), and the number of crypts increases during repair after colonic mucosal injury (18). As the ability to form multilobed organoids is considered a direct stem cell function, the frequency of organoid formation in serial replating experiments serves as a quantitative measurement of ISC function (19). In addition, the Wnt signaling pathway plays an important role in maintaining and regulating ISC self-renewal (20).

The intestinal environment induced by a high-iron diet may adversely affect epithelial cell repair or barrier recovery after injury (5). We hypothesized that high-dose iron aggravates intestinal injury and is detrimental to repair by limiting intestinal epithelial cell renewal and ISC activity. Based on this hypothesis, adult mice with DSS-induced colitis were used as model animals in this study to determine the intestinal index, intestinal morphology, intestinal cell renewal, organoid formation rate, and expression of Wnt target genes as a method to study the intestinal injury induced by the high dose of iron with DSS and to evaluate the repair effect of high-dose iron on the injured intestine. We were surprised to find that high-dose iron increased intestinal inflammation in mice with colitis but promoted intestinal repair.

## Materials and Methods

### Animals and Experimental Procedures

The experimental protocol was reviewed and approved (Approval number 2016-093) by the Animal Care and Use Committee of Hunan Normal University, Changsha City, Hunan, China (21). Seventy-two adult male C57BL/6 mice (aged 8 weeks, similar body weight) were acclimated for 1 week with free access to standard mouse chow and tap water under controlled temperature (23°C), humidity (55% ± 10%), and light (12:12-h light–dark cycle) conditions. The mice were randomly divided into two dietary treatment groups (n = 36): the basal diet supplemented with 45 (control) and 450 mg/kg iron (high-iron) from ferrous sulfate. The mice were fed dietary iron for 2 weeks and administered 2.5% dextran sodium sulfate (DSS, MB5535, Meiluobio Consultancy) in drinking water for 7 days. We found that the survival curves, body weights, and feed intakes of the adult mice decreased substantially during DSS induction, and the mice showed signs of illness, as shown in Supplementary Figure S1. The adult mice were euthanized by isoflurane anesthetization followed by cervical dislocation, and samples of different tissues were collected on day 0 (day 21), 3 (day 24), and 7 (day 28) post administration (DPA).

### Sample Collection and Measurement

**Figure 1:**
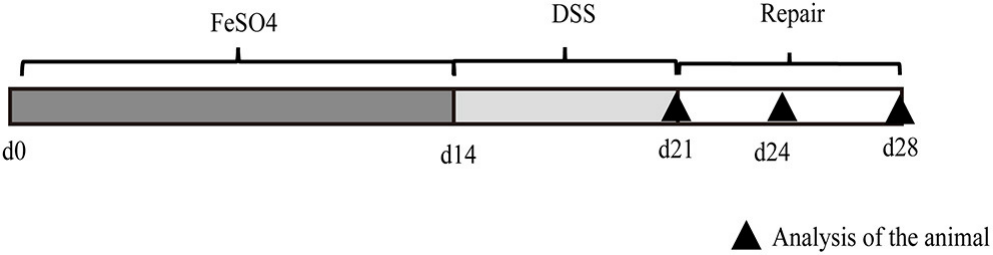
Representative image of the experimental protocol used for the induction of ulcerative colitis in mice. Mice received normal iron content or high-iron diet from ferrous sulfate for 2 weeks (d1–d14) (*n* = 36 for each group). From days 15 to 21, mice were treated with sodium dextran sulfate (DSS) (2.5% DSS in drinking water) and euthanized after DSS withdrawal (d21), or 3 days (d24) and 7 days (d28) after DSS withdrawal (*n* = 12).

The experimental protocol used to induce ulcerative colitis was performed as previously described (22) and is briefly shown in Figure 1A. At 0 DPA, 3 DPA, and 7 DPA, 12 adult mice in each group were euthanized, their small and large intestines were removed, and the intestinal lengths were measured in parallel with a straight edge after the mesentery was removed. After the mesentery and fat were removed, the large and small intestines were weighed separately (23). The intestinal tissues of the jejunum and the mid-colon (approximately 2 cm each) were separated with sterile instruments and washed with phosphate-buffered saline (PBS; 137 mM NaCl, 2.7 mM KCl, 4.3 mM Na_2_HPO_4_, and 1.4 mM KH_2_PO_4_, pH = 7.4). Each fragment (approximately 2 cm long) was fixed with 4% formaldehyde in phosphate buffer and stored at 4°C until the microscopic evaluation of the intestinal morphology and renewal of intestinal epithelial cells (24).

### Examination of Intestinal Morphology

According to the standard paraffin-embedding technique, the intestinal tissues of the jejunum and colon were embedded and fixed, and the embedded wax block was fixed on a microtome and cut into 4 μm-thick sections, and the slices were spread in 42°C water, flattened, and then mounted on glass slides. After the water was drained, the slides were placed on a 37°C baking sheet, stained with hematoxylin and eosin, and inspected under an optical microscope (DM3000; Leica). The villus height and crypt depth of the jejunum, as well as the crypt depth of the colon, were measured at 40× magnification using an image processing and analysis system (Image-Pro Plus version 6.0, Media Cybernetics, San Diego, CA, USA), and the intestinal villus height and crypt depth were measured at five positions (five bright fields with two to three villi per field under the microscope) in each section. The mean villus height and crypt depth in each section of each mouse was then calculated and subjected to further analyses.

### Immunohistochemical Staining for Ki-67, Lysozyme (LYZ), and Sex-Determining Region Y-Box Transcription Factor 9^+^ (SOX-9)

After the slides were placed at 37°C overnight, they were transferred into a water-bath slide dryer at 65°C for 90 min on the next day, dewaxed twice for 10 min each, and then rehydrated with a decreasing series of ethanol solutions, starting with 100% ethanol and decreasing in 5-min intervals to 95 to 85% ethanol and finally to 75% ethanol. We applied 3% hydrogen peroxide (H_2_O_2_) to inactivate endogenous peroxidases and incubated the sections in the dark for 10 min. Antigen retrieval was performed by boiling the samples twice in sodium citrate buffer (0.01 M, pH 6.0). Bovine serum albumin (BSA; 5%; Boster Biological Technology Co., Ltd., Wuhan, China) was used to block nonspecific binding by incubating the sections at 37°C for 40 min at a dilution of 1:10. Sections were then incubated with a Ki-67 antibody (Abcam, ab15580; 1:600 dilution), lysozyme (LYZ) antibody (Abcam, ab108508; 1:700 dilution), or SOX-9 antibody (Millipore, AB5535-25UG; 1:1000 dilution) at 37°C for 90 min. Paneth cells only exist in the crypts of the jejunum and are not found in the colon (16, 25); thus, the LYZ antibody was only incubated with the jejunum sections. Then, the slices were treated with an enzyme-labeled goat anti-rabbit IgG secondary antibody (ZSGB-BIO, Beijing, China) at 37°C for 45 min. Except for the blocking step, each step was followed by four washes with PBS for 3 min each. The slices were treated with a diaminobenzidine (DAB) kit (ZSGB-BIO, Beijing, China) in the dark for 50 s to visualize the positive cells. Microscopic images of intestinal samples from the jejunum and colon of each animal were photographed with an optical microscope (Leica DM3000, Leica Microsystems, Wetzlar, Germany) at 40× magnification. The numbers of cells positive for Ki-67 (colon and jejunum crypts), LYZ (jejunum crypts), and SOX-9 (colon and jejunum crypts) in atleast 10 crypts from each sample were counted using Image-Pro Plus 6.0 software (26).

### Alcian Blue–Periodic Acid Schiff Staining

Tissue sections were stained with Alcian blue–periodic acid Schiff (AB–PAS) (Nanjing Jiancheng Bioengineering Institute, Nanjing, China) according to the manufacturer's protocol. After the slices were dewaxed, they were hydrated in a gradient ranging from 95% ethanol to distilled water, remaining in each gradient solution for 2 min. Alcian blue dye solution was added and stained the sections for 15 min, and then the sections were incubated with the periodate dye solution for 10 min. After washing the sections with distilled water for 2 min, the sections were completely dried, and Schiff reagent was added and incubated with the section for 5 min. Next, the sections were slowly rinsed with running tap water for 5 min. After the sections on the glass slides were air-dried naturally, they were covered with a cover glass for inspection under an optical microscope. Image-Pro Plus version 6.0 software was used to count the number of goblet cells in the intact jejunal villi and crypts of the jejunum and colon and calculate the average values of no <10 counts for each sample (27).

### Counting of Organoids

Adult C57BL/6 mice in the high-iron group and the control group were euthanized at three time points, and an intestinal segment of approximately 6 cm was removed from the anterior jejunum of each mouse. The jejunum was washed with cold PBS, scraped, cut into small pieces, and then incubated with 2.5 mmol/L ethylenediaminetetraacetic acid disodium salt (Sigma–Aldrich, St. Louis, MO, USA) for 30 min and placed on a 4°C rotator for epithelial separation. Next, the sample was washed with PBS until a high crypt purity was achieved and then filtered through a 70-μm cell strainer. Ten percent (vol/vol) fetal calf serum (FCS) was added to the crypt suspension and centrifuged at 1200 rpm for 5 min. The pellet was washed with 2 mL of DMEM-GF (Gibco, Grand Island, NY), and the pelleted crypts were embedded in 8 μL of Matrigel (Corning, Bedford, OH, USA) in a prewarmed 96-well culture dish, with approximately 100 crypts per well. After Matrigel solidification, a culture medium (130 μL) was added. The composition of the medium was described by Wang Z (28). Finally, the organoid formation efficiency was calculated (29).

### Extraction of RNA and Real-Time Quantitative Polymerase Chain Reaction (PCR)

Tissue from each sample was pulverized in liquid nitrogen. Total RNA was extracted from jejunal and colonic samples using RNAiso Plus (TaKaRa, Dalian, China) and then treated with DNase I (TaKaRa, Dalian, China) to remove trace DNA. The RNA was reverse-transcribed (RT) to cDNAs according to the manufacturer's instructions (23). Primer 5.0 (Premier Biosoft International, Palo Alto, California, USA) was used to design the primers used in this study. The selected gene primer sequences are shown in Supplementary Table S1. The cDNAs were diluted (1:4) with sterile double-distilled (dd) water (H_2_O) before they were subjected to real-time quantitative PCR. Each PCR was performed in triplicate. Real-time quantitative PCR analysis was performed with a QuantStudio 5 Real-Time PCR System (Thermo Fisher Scientific Inc., Rockford, IL, USA) in a reaction volume of 10 μL containing 5 μL of SYBR Green mix (TaKaRa, Dalian, China), 1 μL of cDNAs, 0.2 μL each of the forward and reverse primers, 0.2 μL of ROX II, and 3.4 μL of dd H_2_O. The mRNA expression abundance (A) of the target gene was normalized using β-actin and calculated as A = 2^−ΔΔCt^ [Ct(β-actin)-Ct(target)] (30).

### Statistical Analysis

All statistical analyses were calculated using SPSS software (version 22.0; IBM Corp., Chicago, IL, USA). Before analysis, the Shapiro–Wilk normality test and Tukey's *t*-test were performed on the data that conformed to a normal distribution. Any value that deviated from the standardized mean by more than three standard deviations was eliminated. Values are presented as the means ± SEM. *P* < 0.05 indicated that the difference was significant, and *P* < 0.01 indicated that the difference was extremely significant. All graphs presented in this study were plotted using GraphPad Prism 6.0 software (GraphPad Inc., San Diego, CA, USA).

## Results

### Gut Index

**Figure 2:**
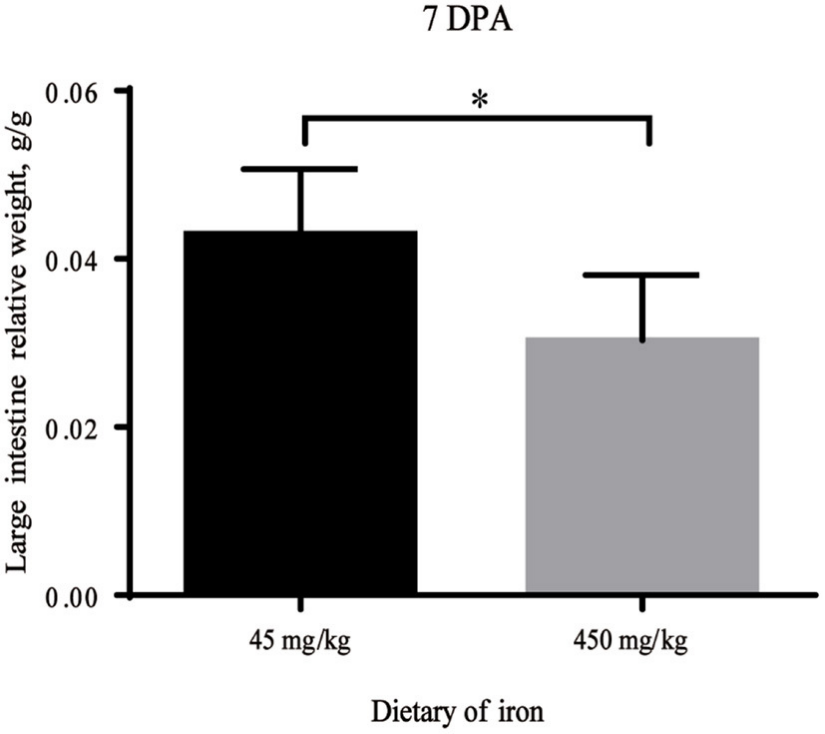
Large intestine relative weight with different levels of ferrous sulfate. * indicates *p* < 0.05, *n* = 12. 7 DPA indicates “day 7 post administration,” which is day 28 of the whole experiment cycle.

As shown in Table 1, no significant differences in the weights of adult mice from the high-iron group and the control group were observed from Day 0 to Day 28 (*P* > 0.05). At 7 DPA, the relative weight of the large intestine was significantly decreased (Figure 2, *P* = 0.035) in the high-iron group. No significant differences (*P* > 0.05) were observed in the intestinal length and weight, relative intestinal length, or relative intestinal weight at 0 DPA (Supplementary Table S2), 3 DPA (Supplementary Table S3), or 7 DPA (Supplementary Table S4).

**Table 1:** The effect of iron in the diet on the body weight of adult mice^1^.

**Items^2^**	**Dietary of iron, mg/kg**		***P*-value**
	**45**	**450**	
**BW, g**			
d 0	20.71 ± 0.15	20.70 ± 0.11	0.964
d 7	21.86 ± 0.14	21.66 ± 0.11	0.268
d 14	23.43 ± 0.14	23.27 ± 0.15	0.461
d 21	19.79 ± 0.30	18.83 ± 0.39	0.070
d 24	19.07 ± 0.61	17.31 ± 0.50	0.069
d 28	25.23 ± 0.29	24.33 ± 2.57	0.750

1 *Body weight changes of adult mice fed a high-iron diet and normal diet from day 1 to day 28. Values are expressed as mean ± SEM; n = 36 (d 0, d 7, d 14), n = 12 (d 21, d 24, d 28); ^2^BW, body weight*.

### Intestinal Morphology

**Figure 3:**
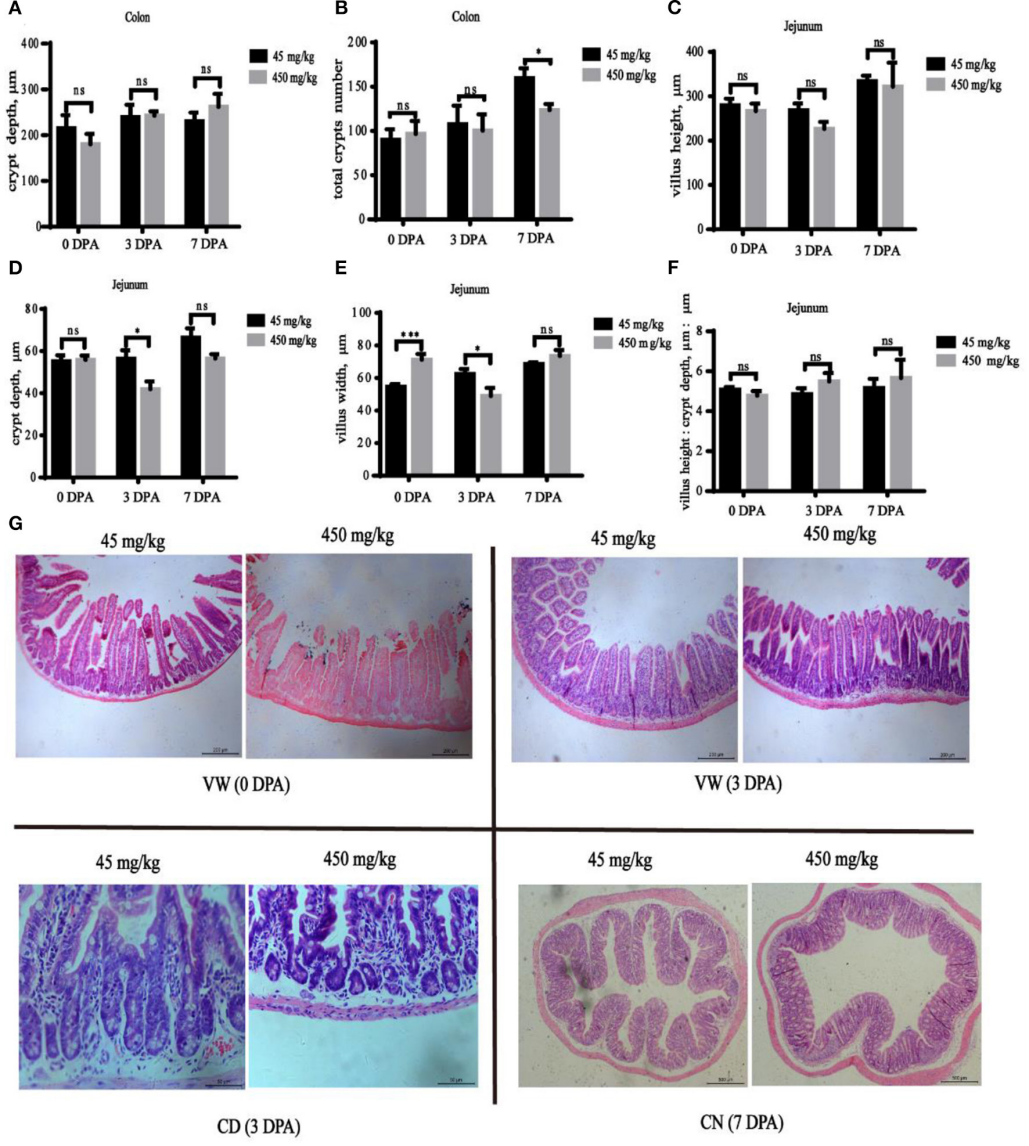
Effects of dietary iron on jejunum and colon injury and repair in adult mice induced by DSS. The effect of high-iron diet on **(A)** the depth of colonic crypts, **(B)** the number of colonic crypts, **(C)** jejunum villus height, **(D)** jejunum crypt depth, **(E)** jejunum villus width, and **(F)** relative height of villus at 0 DPA, 3 DPA, and 7 DPA (*n* = 12), ns means no significance, * indicates *p* < 0.05, while **indicates *p* < 0.01, and ***indicates *p* < 0.001. **(G)** At 0 DPA, 3 DPA, and 7 DPA, HE staining intestinal morphology images of jejunum and colon in the high-iron group and the control group. VW indicates “Villus Width,” CD indicates “Crypt Depth,” and CN indicates “Crypts Number.” 0 DPA indicates “day 0 post administration,” which is day 21 of the whole experiment cycle; 3 DPA indicates “day 3 post administration,” which is day 24 of the whole experiment cycle; 7 DPA indicates “day 7 post administration,” which is day 28 of the whole experiment cycle. Note Scale bars, VW is 200 μm (magnification 100×), CD is 50 μm (magnification 400×), CN is 500 μm (magnification 50×).

The jejunum villus width was significantly increased (Figure 3E, *P* < 0.001) in the high-iron group at 0 DPA, and the jejunum villus width (Figure 3E, *P* = 0.033) and jejunum crypt depth (Figure 3D, *P* = 0.031) at 3 DPA and the number of colonic crypts (Figure 3B, *P* = 0.042) at 7 DPA were significantly decreased in the high-iron group. However, significant differences (*P* > 0.05) in the colonic crypt depth (Figure 3A), jejunum villus height (Figure 3C), or villus height: crypt depth (Figure 3F) were not observed at the three time points. Representative images of intestinal morphology are shown in Figure 3G.

### Renewal Status of Intestinal Epithelial Cells

**Figure 4:**
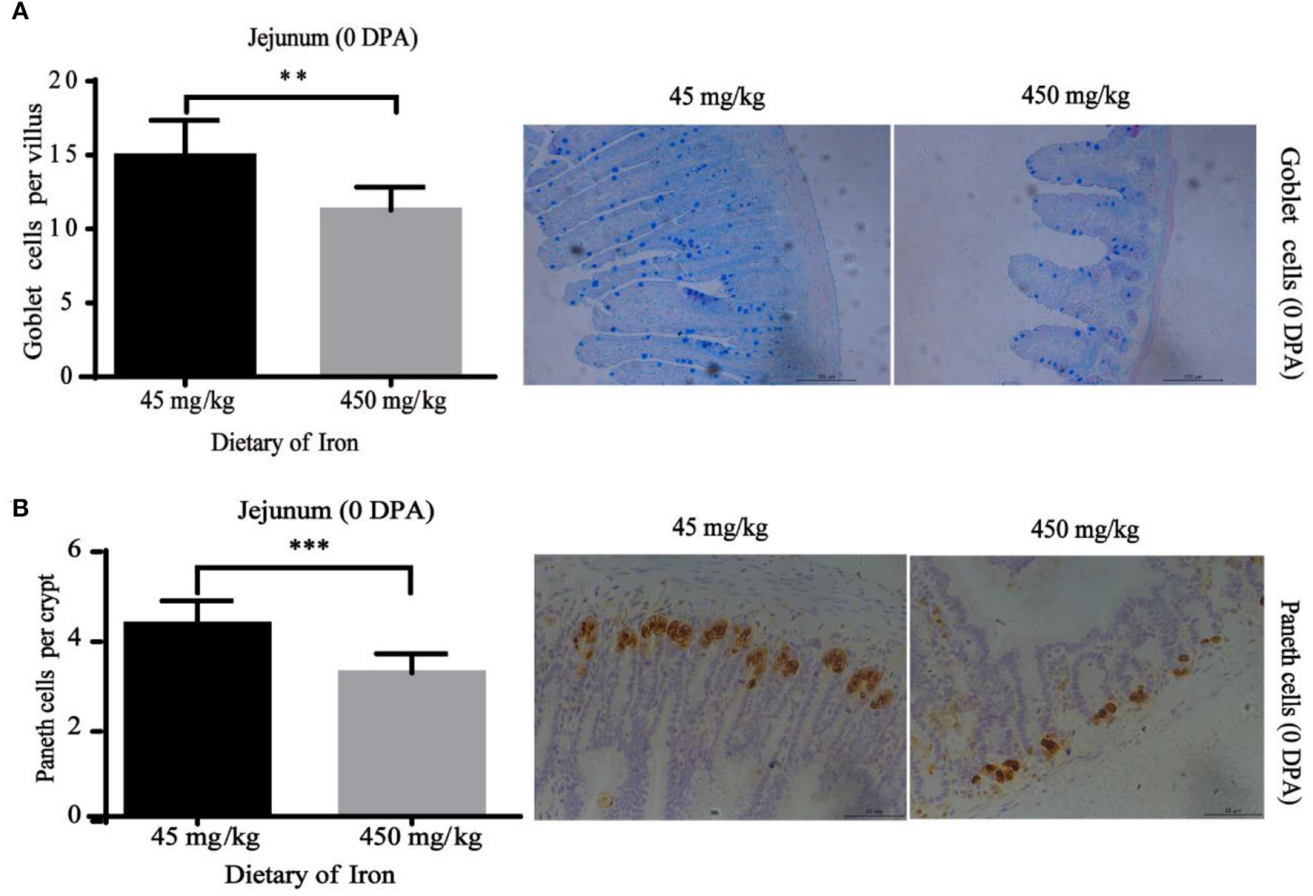
Effects of dietary iron on intestinal epithelial cell renewal in adult mice at 0 DPA. **(A)** Goblet cell number of jejunum villi **(B)** Paneth cells number in jejunum crypt. *n* = 12. 0 DPA indicates “day 0 post administration,” which is day 21 of the whole experiment cycle. Note Scale bars. A used 100 μm (magnification 200×), B used 50 μm (magnification 400×). **indicates *p* < 0.01, and ***indicates *p* < 0.001.

**Figure 5:**
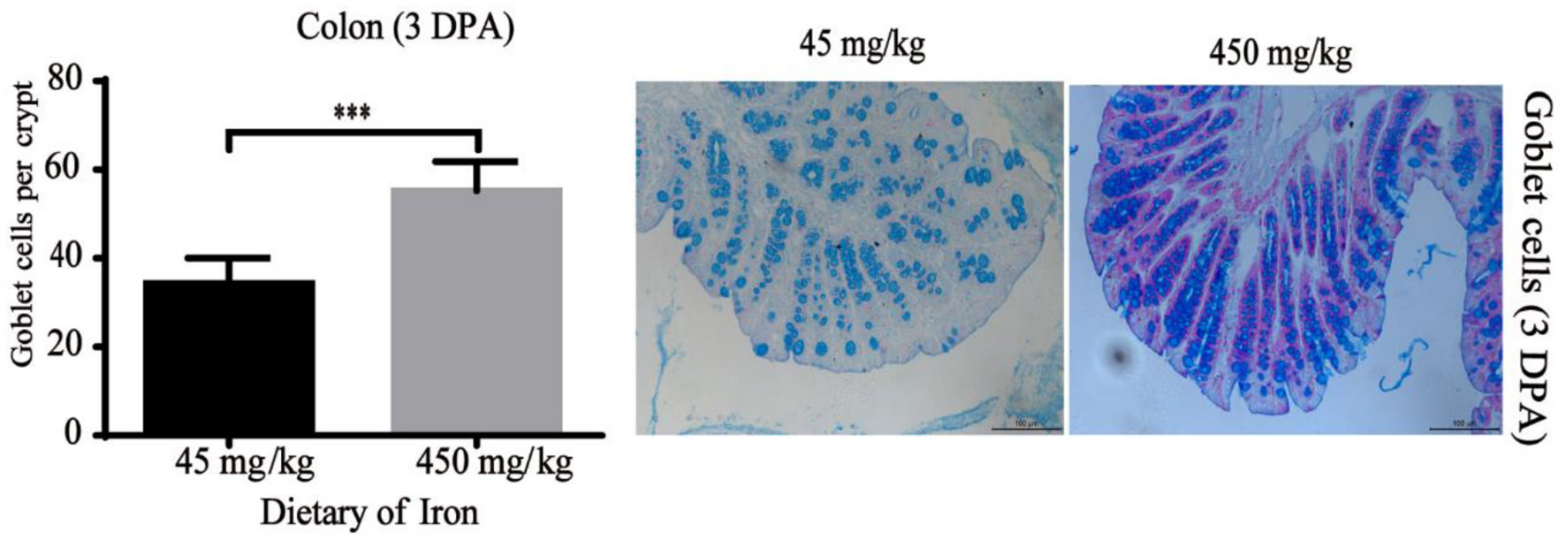
Effects of dietary iron on intestinal epithelial goblet cell renewal in adult mice at 3 DPA. The number of goblet cells in the colonic crypt, *n* = 12. The 3 DPA indicates “day 3 post administration,” which is day 24 of the whole experiment cycle. Note Scale bars, 100 μm (magnification 200×), ^***^ indicates *p* < 0.001.

**Figure 6:**
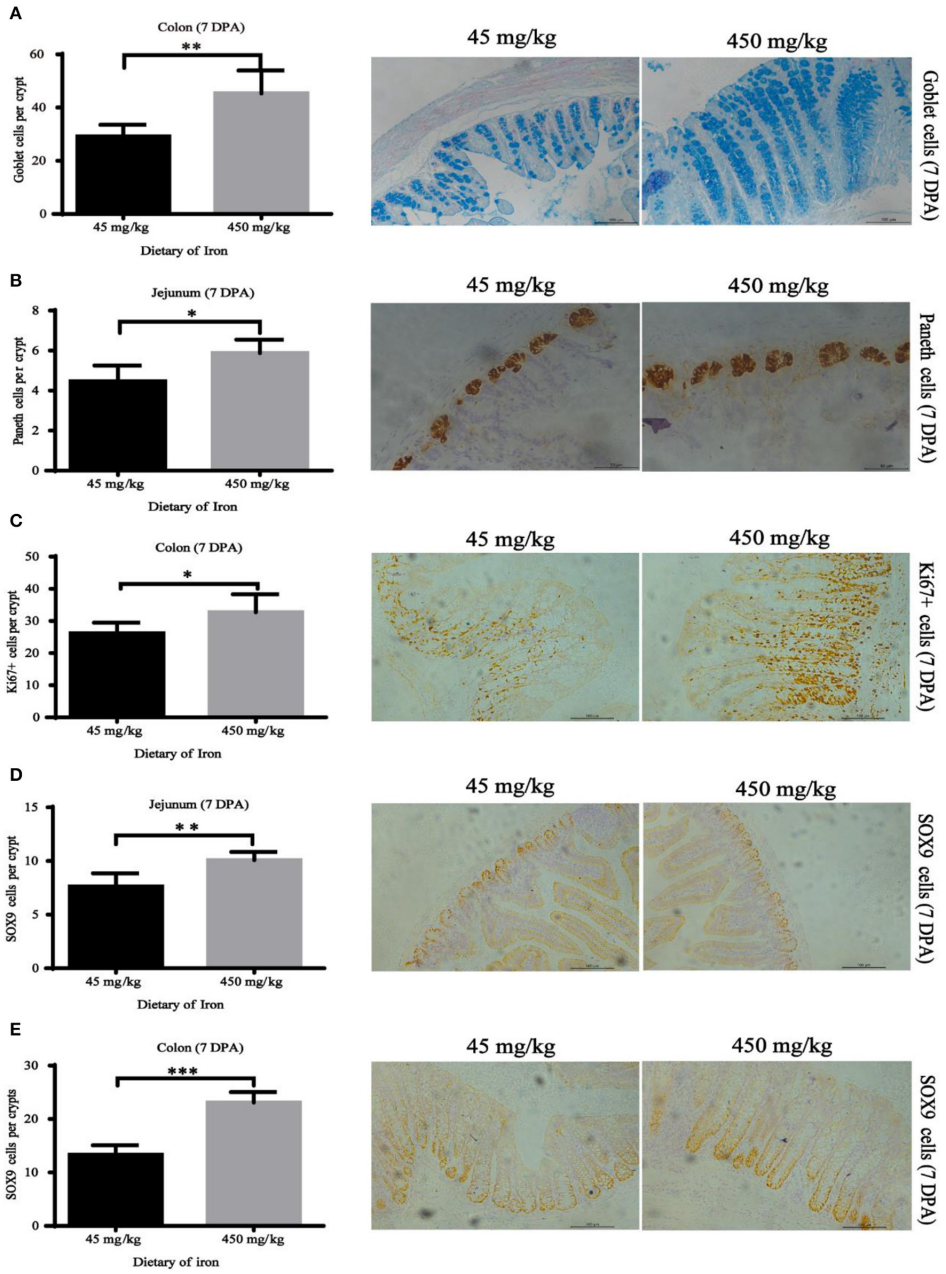
Effects of dietary iron on regeneration of intestinal epithelial cells in adult mice at 7 DPA. **(A)** Goblet cells in colonic crypt, **(B)** Paneth cells in jejunum crypt, **(C)** Ki67 cells in colonic crypt, **(D)** SOX9 cells in jejunum crypt, and **(E)** SOX9 cells in the colonic crypt. *n* = 12. The 7 DPA indicates “day 7 post administration,” which is the day 28 of the whole experiment cycle. Note Scale bars, B used 50 μm (magnification 400×), the rest used 100 μm (magnification 200×). * indicates *p* < 0.05, ** indicates *p* < 0.01, and *** indicates *p* < 0.001.

The numbers of goblet cells in the jejunal villi (Figure 4A, *P* = 0.003) and Paneth cells in the jejunal crypts (Figure 4B, *P* < 0.001) were substantially decreased in the high-iron group at 0 DPA. However, the number of goblet cells in the colon crypt (Figure 5, *P* < 0.001) was substantially increased in the high-iron group at 3 DPA. At 7 DPA, numbers of goblet cells in the colon crypts (Figure 6A, *P* = 0.004), Paneth cells in the jejunum crypts (Figure 6B, *P* = 0.020), Ki67-positive proliferating cells in the colon (Figure 6C*, P* = 0.046), and SOX9-positive cells in the jejunum crypts (Figure 6D*, P* = 0.008) and colon (Figure 6E*, P* < 0.001) were significantly increased.

### Organoid Formation

The organoid formation rates of samples from adult mice in the high-iron group were significantly higher than those in the control group at 3 DPA (Table 2, *P* < 0.001) and 7 DPA (Table 2, *P* < 0.001).

**Table 2:** Effects of dietary iron on organoid formation rate of adult mice after repair^1^.

**Organoid formation(%)**	**Dietary of iron, mg/kg**		***P-*value**
	**45**	**450**	
3 DPA	2.36 ± 0.45	16.94 ± 1.85	<0.001
7 DPA	22.14 ± 1.20	58.93 ± 2.77	<0.001

1 *Adult mice fed diets containing 45 mg/kg and 450 mg/kg iron from ferrous sulfate for 2 weeks and then DSS was orally administrated to all mice for 7 days, isolated organoids from jejunum at days 3 and 7 post administration (DPA), and observed the organoid formation rate. Values are expressed as mean ± SEM, n = 6*.

### Expression of Genes Related to the Wnt Pathway

As shown in Table 3, the mRNA expression of Wnt/β-catenin target genes, such as jagged canonical Notch ligand 2 (*Jag-2*), was downregulated in the jejunal tissue from the high-iron group at 0 DPA (*P* = 0.007) compared with the control group. Serum/glucocorticoid-regulated kinase 1 (*Sgk1*) expression was upregulated in the colon of the high-iron group at 3 DPA (*P* = 0.017). However, bone morphogenetic protein 4 (*Bmp4*) (*P* = 0.015) and neural precursor cell-expressed developmentally downregulated 8 (*Nedd8*) (*P* = 0.007) expression in the jejunum, *Bmp4* (*P* = 0.022), and *Nedd8* (*P*= 0.014) expressions in the colon were downregulated in the high-iron group at 7 DPA. In addition, severe inflammation may have led to lower mRNA levels in the colon at 0 DPA, which affected our measurements; thus, we did not provide the corresponding data in this paper.

**Table 3:** Effects of dietary iron on Wnt target gene in adult mice^1^.

**Items^**2**^**	**Dietary of iron, ppm**		***P-*value**
	**45**	**450**	
**Jejunum (0 DPA)**			
*Bmp4*	1.02 ± 0.09	1.13 ± 0.14	0.527
*Jag-1*	1.02 ± 0.09	0.84 ± 0.10	0.221
*Nedd8*	1.04 + 0.12	1.14 + 0.10	0.530
*Sgk-1*	1.02 ± 0.10	0.89 ± 0.22	0.623
*Ephb4*	1.05 ± 0.13	0.79 ± 0.16	0.251
*Jag-2*	1.02 ± 0.08	0.54 ± 0.12	0.007
*Edn3*	2.05 ± 1.03	6.07 ± 3.71	0.522
**Jejunum (3 DPA)**			
*Bmp4*	1.04 ± 0.11	0.98 ± 0.11	0.720
*Jag-1*	1.48 ± 0.38	1.26 ± 0.11	0.676
*Nedd8*	1.04 ± 0.11	1.07 ± 0.11	0.870
*Sgk-1*	1.59 ± 0.30	2.44 ± 0.40	0.110
*Ephb4*	1.01 ± 0.23	1.18 ± 0.21	0.611
*Jag-2*	0.93 ± 0.10	0.70 ± 0.34	0.086
*Edn3*	2.23 ± 1.08	1.15 ± 0.42	0.461
**Jejunum (7 DPA)**			
*Bmp4*	1.01 ± 0.05	0.74 ± 0.07	0.015
*Jag-1*	1.09 ± 0.19	0.89 ± 0.16	0.433
*Nedd8*	1.02 ± 0.10	0.57 ± 0.04	0.007
*Sgk-1*	1.30 ± 0.43	0.87 ± 0.16	0.457
*Ephb4*	1.33 ± 0.96	1.00 ± 0.17	0.541
*Jag-2*	1.53 ± 0.55	1.40 ± 0.24	0.863
*Edn3*	1.50 ± 0.69	1.33 ± 0.66	0.925
**Colon (3 DPA)**			
*Bmp4*	1.13 ± 0.24	0.72 ± 0.07	0.235
*Jag-1*	1.07 ± 0.16	1.55 ± 0.93	0.056
*Nedd8*	1.07 ± 0.16	1.09 ± 0.11	0.944
*Sgk-1*	1.19 ± 0.28	3.54 ± 0.99	0.017
*Ephb4*	1.70 ± 0.90	3.33 ± 0.74	0.089
*Jag-2*	1.62 ± 0.68	2.21 ± 0.55	0.574
*Edn3*	1.11 ± 0.21	0.65 ± 0.14	0.158
**Colon (7 DPA)**			
*Bmp4*	1.06 ± 0.09	0.69 ± 0.08	0.022
*Jag-1*	0.95 ± 0.12	0.96 ± 0.10	0.921
*Nedd8*	1.03 ± 0.18	0.58 ± 0.04	0.014
*Sgk-1*	1.00 ± 0.28	1.74 ± 0.34	0.134
*Ephb4*	0.92 + 0.19	1.46 ± 0.18	0.086
*Jag-2*	1.15 ± 0.32	1.87 ± 0.22	0.116
*Edn3*	2.16 ± 0.95	1.51 ± 0.40	0.556

1 *The values are expressed as mean ± SEM (n = 12). Differences were assessed by the Student's t-test. Values of P < 0.05 are referred to as statistically significant. ^2^Bmp4, bone morphogenetic protein 4; Jag1, jagged canonical Notch ligand 1; Nedd8, neural precursor cell expressed developmentally down-regulated 8; Sgk1, serum/glucocorticoid regulated kinase 1; Ephb4, EPH receptor B4; Jag2, jagged canonical Notch ligand 2; Edn3, endothelin 3. 0 DPA indicates “day 0 post administration,” which is day 21 of the whole experiment cycle. 3 DPA indicates “day 3 post administration,” which is day 24 of the whole experiment cycle. 7 DPA indicates “day 7 post administration,” which is day 28 of the whole experiment cycle*.

## Discussion

This study was conducted to investigate the effects of high dietary iron supplementation on intestinal injury and intestinal repair function, especially for ISCs in the jejunum and colon of adult mice. The symptoms of DSS-induced colitis are weight loss (31), disruption of the gut index (32) and morphology, and damage to intestinal stem cells (33). *In vitro* organoid cultures were used to observe the regulation of ISC compartments after tissue injury (34). Furthermore, the Wnt/β-catenin pathway is essential for intestinal renewal and ISC maintenance (35). Disease phenotypes were studied according to body weights and intestinal indices (intestinal length, intestinal weight, intestinal length:body weight, and intestinal weight:body weight). In addition, histological parameters (villus width, villus height, crypt depth, villus height:crypt depth, and crypt number), intestinal epithelial cell renewal, the organoid formation rate, and the expression of related target genes in the Wnt pathway were assessed.

The regeneration of intestinal epithelial cells increased in the high-iron group at 7 DPA, while the relative weight of the large intestine was reduced, which may have been related to the decreased number of colonic crypts. Liang, L et al. (36) reported that iron supplementation increased colon weight and cell (goblet cell) renewal. These inconsistent results may be due to differences in the dietary mode or iron concentration. Oral iron supplementation with 450 mg/kg ferrous sulfate was used in our study, while Liang, L et al. administered an intraperitoneal injection of 120 μg/kg iron–dextran.

The intestinal function is closely related to intestinal tissue morphology. Pereira et al. (37) reported that during inflammatory states, villi often become wider, although they tend to become shorter, which explains the maintenance of the intestinal epithelial cell count. As shown in our study, the villus width increased in the high-iron group at 0 DPA, but no significant difference in villus height was observed. The morphological changes suggest an effect of high-dose iron on intestinal function. Furthermore, the villus width and crypt depth in the jejunum decreased at 3 DPA. The decrease in villus width in the high-iron group may be related to the decreased crypt depth at 3 DPA. According to the study by Holle, GE (38), the proliferative zone in the crypt expanded in proportion to the total crypt depth. However, the results of studies examining the effects of a high iron concentration on the morphology of the small intestine are contradictory. One possible explanation for this difference is the complexity of the intestinal repair.

We determined whether morphological changes observed during intestinal injury and repair are caused by altered epithelial cell renewal induced by dietary supplementation with high-dose iron by calculating the numbers of Ki67-positive, Paneth, and Sox9-positive cells in the crypt base and goblet cells in crypts and villi. Ki67, a marker of proliferating cells, labels undifferentiated proliferating transit-amplifying (TA) cells (progenitors) in the crypt, which are derived from ISCs and differentiate into functional epithelial cells (39, 40). We found that Ki67 levels in the high-iron group were increased significantly at 7 DPA, suggesting that the proliferation of intestinal epithelial cells was increased. SOX9 is expressed in ISCs to regulate ISC proliferation and differentiation (41). The number of SOX9 cells in the high-iron group was increased at 7 DPA, indicating increased ISC activity. Goblet cells and Paneth cells are secretory cells (39). Paneth cells produce abundant antibacterial peptides/proteins that confer mucosal protection and provide signals for the maintenance of ISCs for normal mucosal renewal. Goblet cells synthesize and release various mucin proteins that are major components of the unstirred mucus layer covering the epithelium (42, 43). The number of goblet and Paneth cells increased in the high-iron group at 3 DPA and 7 DPA. In contrast, the numbers of both types of cells were decreased in the high-iron group at 0 DPA. Based on these findings, high-dose iron exerts an adverse effect on the intestinal tract after injury but exerts protective effects on intestinal barrier function and epithelial homeostasis after repair. Notably, the markers Ki67 and SOX9 were not detected in the intestinal tissues of adult mice at 3 DPA, which was speculated to be related to severe intestinal damage.

ISCs, which are located at the bases of crypts, are responsible for intestinal epithelial cell self-renewal and intestinal epithelial homeostasis throughout the lifespan of an organism (44), and ISCs generated from crypts develop into organoids *in vitro*, simulating the ISC niche *in vivo* (45). Organoid budding may be similar to the expansion of the ISC compartment and the formation of new crypts through crypt fission (46). We isolated and cultured jejunal organoids from adult mice in the high-iron group and the control group to test the effects of high-dose iron on intestinal epithelial homeostasis. Intestinal injury after DSS induction was serious and markedly altered the formation of organoids at 0 DPA. In addition, the organoid formation rates of mice in the high-iron group were higher than those of mice in the control group at 3 DPA and 7 DPA. These findings suggest that high iron levels modulate ISC activity after repair and further validate that a high iron concentration promotes intestinal epithelial renewal.

The Wnt signaling pathway is critical for maintaining and regulating ISCs (20). We explored the potential mechanisms of ISC differentiation and self-renewal by detecting the expression of genes related to the Wnt signaling pathway *in vitro*. The Wnt/β-catenin pathway is essential for ISC self-renewal, and β-catenin-target genes such as *Bmp4, Jag1, Jag2, Ephb4, Nedd8, Edn3*, and *Sgk1* are expressed to maintain ISC and progenitor cell proliferation (21). The Jag-2 signaling pathway is involved in early epithelial regeneration after intestinal injury by promoting crypt epithelial cell proliferation (47). The expression of *Jag-2* was downregulated in mice fed with high-dose iron at 0 DPA, suggesting that high-dose iron inhibited intestinal epithelial cell regeneration by reducing *Jag-2* expression. Sgk1 exerts an anti-apoptotic effect (48), and the upregulation of *Sgk1* expression in the high-iron group at 3 DPA may indicate a positive effect on the intestinal epithelium. According to some studies, *Bmp4* is a proinflammatory gene that induces endothelial dysfunction and aggravates tissue damage (49). In addition, *Nedd8* is also associated with the inflammatory response, and the downregulation of *Nedd8* expression inhibits NF-κB phosphorylation (NF-κB is a ubiquitous proinflammatory transcription factor in mammalian cells), thereby reducing inflammation (50). In our study, inflammation in the jejunum and colon was reduced by the downregulation of *Bmp4* and *Nedd8* expression levels in the high-iron group at 7 DPA.

Based on these results, a high-iron diet aggravates intestinal injury in adult mice but exerts a positive effect on the intestinal repair. This finding seems to contradict our hypothesis that high iron concentrations are not beneficial to intestinal repair. However, it may provide new insight into the postoperative repair of colitis in livestock and poultry production.

## Data Availability Statement

The original contributions presented in the study are included in the article/supplementary material, further inquiries can be directed to the corresponding author/s.

## Ethics Statement

The animal study was reviewed and approved by Animal Care and Use Committee of Hunan Normal University, Changsha City, Hunan, China.

## Author Contributions

YZ: investigation, data curation, and writing—original draft preparation. LY: conceptualization, methodology, software, and writing—review and editing. XZ: visualization and investigation. JL: investigation. YY: resources. QW: conceptualization and methodology. JL: project administration. HY: supervision, funding acquisition, and writing—review and editing. All authors contributed to the article and approved the submitted version.

## Funding

This work was supported by the National Natural Science Foundation of China (Grant No. 32130099), the Hunan Province's Changsha_Zhuzhou_Xiangtan National Independent Innovation Demonstration Zone projects (Grant No. 2017XK2058), and the Hunan Provincial Key Laboratory of Animal Nutritional Physiology and Metabolic Process open fund projects (Grant No. ISA2020113).

## Conflict of Interest

JL and HY were employed by Fujian Aonong BiologicaI Science and Technology Group Co., Ltd. The remaining authors declare that the research was conducted in the absence of any commercial or financial relationships that could be construed as a potential conflict of interest.

## Publisher's Note

All claims expressed in this article are solely those of the authors and do not necessarily represent those of their affiliated organizations, or those of the publisher, the editors and the reviewers. Any product that may be evaluated in this article, or claim that may be made by its manufacturer, is not guaranteed or endorsed by the publisher.
