# Supplementary material for: Dietary High Dose of Iron Aggravates the Intestinal Injury but Promotes Intestinal Regeneration by Regulating Intestinal Stem Cells Activity in Adult Mice With Dextran Sodium Sulfate-Induced Colitis
Source: Front Vet Sci. 2022 Jun 15;9:870303. doi: 10.3389/fvets.2022.870303 (PMC9240710; doi:10.3389/fvets.2022.870303)
Supplement: Supplementary file 1 [file Table_1.DOCX]

Supplementary Material

**Table S1.** Primers used for real-time PCR analysis

| **Wnt/β-catenin target genes (Jejunum and Colon)^1^** | **Primers** | **Sequence (5’-3’)** | **Products length, bp** |
| --- | --- | --- | --- |
| *Bmp4* | Forward | AACCAACCATGCCATTGTGC | 91 |
|  | Reverse | AATGGCACTCAGTTCAGTGG |  |
| *Jag-1* | Forward | AAGTCCGCTTTGCCAAACAG | 92 |
|  | Reverse | TGTCCAGTTCGGGTGTTTTG |  |
| *Nedd8* | Forward | AAAGTGAAGACGCTGACTGG | 88 |
|  | Reverse | TTTCTTCCACACGCTCCTTG |  |
| *Sgk-1* | Forward | TGCATGCAAACACGCTGAAG | 107 |
|  | Reverse | TTTGTTGAGAGGGACTTGGC |  |
| *Ephb4* | Forward | TCGGATAAGCATGGGCAGTATC | 101 |
|  | Reverse | TTGGCAAATTCCCTCACTGC |  |
| *Jag-2* | Forward | TTGATGAGTGTGCCTCTAACCC | 150 |
|  | Reverse | AAGCATTAAGGCACGGCTTC |  |
| *Edn3* | Forward | ATTGTGTCCCCAACAGTTGC | 139 |
| *β-actin* | Reverse  Forward  Reverse | TGTAAGTGAAGCACGTGCAG  CATTGCTGACAGGATGCAGAAGG  TGCTGGAAGGTGGACAGTGAGG | 138 |

^1^*Bmp4* = bone morphogenetic protein 4; *Jag1* = jagged canonical Notch ligand 1; [*Nedd8*](https://www.sciencedirect.com/topics/agricultural-and-biological-sciences/precursor-cell) = neural precursor cell expressed developmentally down-regulated 8; [*Sgk1*](https://www.sciencedirect.com/topics/agricultural-and-biological-sciences/glucocorticoid) = serum/glucocorticoid regulated kinase 1; *Ephb4* = EPH receptor B4; *Jag2* = jagged canonical Notch ligand 2; *Edn3* = endothelin 3.

**Table S2.** Effects of dietary iron on intestinal index of adult mice after DSS induction¹

| **Items^²^** | **Dietary of Iron, mg/kg** | | ***P*-value** |
| --- | --- | --- | --- |
|  | **45** | **450** |  |
| **Small intestine** |  |  |  |
| Total length, cm | 33.70±0.68 | 33.81±0.74 | 0.912 |
| Total weight, g | 0.7925±0.020 | 0.8084±0.043 | 0.744 |
| Relative length, cm/g | 1.75±0.04 | 1.76±0.05 | 0.817 |
| Relative weight, g/g | 0.0411±0.001 | 0.0421±0.002 | 0.727 |
| **Large intestine** |  |  |  |
| Total length, cm | 5.53±0.14 | 5.81±0.11 | 0.126 |
| Total weight, g | 0.4735±0.030 | 0.4287±0.030 | 0.310 |
| Relative length, cm/g | 0.29±0.01 | 0.30±0.01 | 0.293 |
| Relative weight, g/g | 0.0246±0.002 | 0.0222±0.001 | 0.306 |

¹Day 15 to 21, adult mice were given 2.5% DSS for 7 days to induce colitis. After induction, 12 adult mice were randomly selected from the high iron group and the control group, sacrificed for sampling and measued intestinal index. Values are expressed as mean ± SEM, n = 12.

²Relative weight = the ratio of total intestine weight to body weight at killing; Relative length = the ratio of total intestine length to body weight at killing.

**Table S3.** Effects of dietary iron on intestinal index of adult mice after 3 days of repair¹

| **Items²** | **Dietary of Iron, mg/kg** | | ***P*-value** |
| --- | --- | --- | --- |
|  | **45** | **450** |  |
| **Small intestine** |  |  |  |
| Total length, cm | 33.94±0.61 | 32.00±1.54 | 0.200 |
| Total weight, g | 0.8018±0.082 | 0.6233±0.041 | 0.118 |
| Relative length, cm/g | 1.81±0.06 | 1.83±0.11 | 0.857 |
| Relative weight, g/g | 0.0445±0.006 | 0.0354±0.002 | 0.192 |
| **Large intestine** |  |  |  |
| Total length, cm | 6.67±0.35 | 6.50±0.42 | 0.758 |
| Total weight, g | 0.6145±0.078 | 0.4576±0.037 | 0.142 |
| Relative length, cm/g | 0.37±0.03 | 0.37±0.03 | 0.911 |
| Relative weight, g/g | 0.0340±0.005 | 0.0260±0.002 | 0.235 |

¹Day 21, DSS solution was withdrawn for all adult mice, 12 adult mice were randomly selected from the high iron group and the control group on day 24 (repair for 3 days), sacrificed for sampling and measued intestinal index, n=12.

²Relative weight = the ratio of total intestine weight to body weight at killing; Relative length = the ratio of total intestine length to body weight at killing.

**Table S4.** Effects of dietary iron on intestinal index of adult mice after 7 days of repair¹

| **Items²** | **Dietary of Iron,** **mg/kg** | | ***P*-value** |
| --- | --- | --- | --- |
|  | **45** | **450** |  |
| **Small intestine** |  |  |  |
| Total length, cm | 34.33±0.95 | 34.38±0.24 | 0.973 |
| Total weight, g | 1.1829±0.060 | 1.1196±0.120 | 0.658 |
| Relative length, cm/g | 1.45±0.10 | 1.34±0.04 | 0.409 |
| Relative weight, g/g | 0.0503±0.005 | 0.0432±0.004 | 0.360 |
| **Large intestine** |  |  |  |
| Total length, cm | 8.25±0.34 | 8.45±0.51 | 0.740 |
| Total weight, g | 1.0230±0.060 | 0.7862±0.107 | 0.069 |
| Relative length, cm/g | 0.35±0.04 | 0.33±0.02 | 0.915 |
| Relative weight, g/g | 0.0430±0.003 | 0.0303±0.004 | 0.035 |

¹Day 28 (repair for 7 days), 12 adult mice were randomly selected from the high iron group and the control group, sacrificed for sampling and measued intestinal index. Values are expressed as mean ± SEM, n =12

²Relative weight = the ratio of total intestine weight to body weight at killing; Relative length = the ratio of total intestine length to body weight at killing.


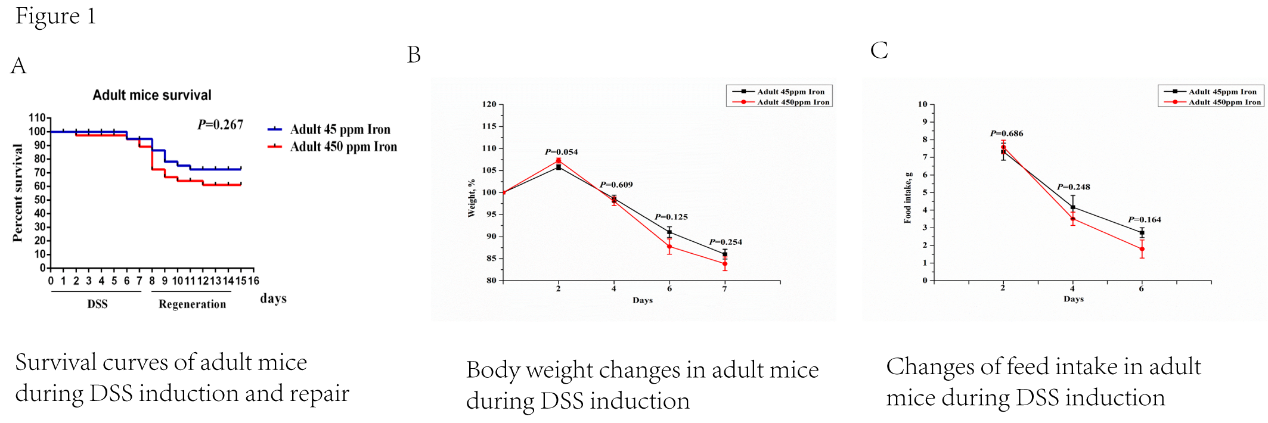


**Figure S1.** Survival curves, body weight, and feed intake of adult mice during DSS induction and repair.
